# Identification of GUCA2A and COL3A1 as prognostic biomarkers in colorectal cancer by integrating analysis of RNA-Seq data and qRT-PCR validation

**DOI:** 10.1038/s41598-023-44459-y

**Published:** 2023-10-10

**Authors:** Seyed Taleb Hosseini, Farkhondeh Nemati

**Affiliations:** 1 Department of Biology, Faculty of Basic Sciences, Qaemshahr Branch, Islamic Azad University, Mazandaran, Iran; 2 Young Researchers and Elite Club, Qaemshahr Branch, Islamic Azad University, Mazandaran, Iran

**Keywords:** Cancer, Computational biology and bioinformatics, Molecular biology

## Abstract

By 2030, it is anticipated that there will be 2.2 million new instances of colorectal cancer worldwide, along with 1.1 million yearly deaths. Therefore, it is critical to develop novel biomarkers that could help in CRC early detection. We performed an integrated analysis of four RNA-Seq data sets and TCGA datasets in this study to find novel biomarkers for diagnostic, prediction, and as potential therapeutic for this malignancy, as well as to determine the molecular mechanisms of CRC carcinogenesis. Four RNA-Seq datasets of colorectal cancer were downloaded from the Sequence Read Archive (SRA) database. The metaSeq package was used to integrate differentially expressed genes (DEGs). The protein–protein interaction (PPI) network of the DEGs was constructed using the string platform, and hub genes were identified using the cytoscape software. The gene ontology and KEGG pathway enrichment analysis were performed using enrichR package. Gene diagnostic sensitivity and its association to clinicopathological characteristics were demonstrated by statistical approaches. By using qRT-PCR, GUCA2A and COL3A1 were examined in colon cancer and rectal cancer. We identified 5037 differentially expressed genes, including (4752 upregulated, 285 downregulated) across the studies between CRC and normal tissues. Gene ontology and KEGG pathway analyses showed that the highest proportion of up-regulated DEGs was involved in RNA binding and RNA transport. Integral component of plasma membrane and mineral absorption pathways were identified as containing down-regulated DEGs. Similar expression patterns for GUCA2A and COL3A1 were seen in qRT-PCR and integrated RNA-Seq analysis. Additionally, this study demonstrated that GUCA2A and COL3A1 may play a significant role in the development of CRC.

## Introduction

Colorectal cancer (CRC) is the second most fatal malignancy worldwide, accounting for around 10% of all cancer-related deaths each year^1,2^. Every year, there are about 1.4 million new incidences of cancer detected, and CRC caused 700,000 mortalities worldwide^3^. Patients with CRC who are detected early stage have a 90% 5-year survival rate, while those who are diagnosed later have a rate of no more than 12%^4,5^. Colon adenocarcinoma, one of the most common types of colorectal cancer, has incidence and death rates of 10.2% and 9.2%, respectively^6,7^. Due to a lack of diagnostic biomarkers and insufficient understanding of the fundamental molecular mechanism, the incidence and mortality of CRC continue to increase^8^. Because of the limits of existing screening technologies and the high metastatic potential of CRC, it is frequently identified at an advanced stage^9^. Detection and monitoring of CRC occurrence and progression are dependent on a combination of radiologic examinations and serum biomarker measurements^10^. In some cases, biomarker levels remain constant and the levels of biomarkers can fluctuate in various disorders^11,12^. Moreover, some patients decide against undergoing a colonoscopy because it is uncomfortable^13^. In the early stages of colon cancer, patients have no specific clinical symptoms^14^. When patients seek medical care, they typically are in the middle or late stages and both the treatment and outlook are poor^14^. Tumor metastasis is the main cause of colon cancer patients’ mortality^15^. Patients suffering from metastatic colon cancer had a considerably lower 5-year survival rate than those with non-metastatic colon cancer^16^. Therefore, it is crucial to choose and identify the specific biomarkers of COAD for early diagnosis, development of a successful treatment plan and the evaluation of patient prognosis^17–19^. Due to their prognostic or predictive potential, circulating carcinoembryonic antigen levels and tumor-associated genes such as APC, KRAS, p53, MSI, SOCS2 and SOCS6 have been proposed as CRC biomarkers^20,21^. Bioinformatics tools have been integrated for numerous diseases including CRC and have the potential to speed up biomarker development^22,23^. Using gene microarray and high throughput sequencing technology researchers have recently examined novel gene expression, therapeutic targets and CRC pathogenesis^24^. The identification of biomarkers for diagnostic and prognostic purposes as well as a better comprehension of the molecular mechanism underlying carcinogenesis may be obtained by the examination of differential expression between cancer and normal cells. RNA Sequencing a beneficial alternative to conventional microarrays has recently become to be used to assess global genomic expressions^25,26^. Previous studies comparing RNA-Seq data with microarray data parallelly have reported that RNA-Seq has advantages over microarray in identifying differentially expressed genes (DEGs) because of greater efficiency and higher resolution^27^. Recently the approach of integrating analysis was created to overcome these difficulties and increase the statistical power for finding DEGs^28^.

In this research we first analyzed the FASTQ file and read count data of the CRC samples that were collected from SRA and TCGA databases and then we validated these in silico findings using samples from 20 Iranian CRC patients.

## Materials and methods

### Identification of RNA-Seq data sets

**Figure 1:**
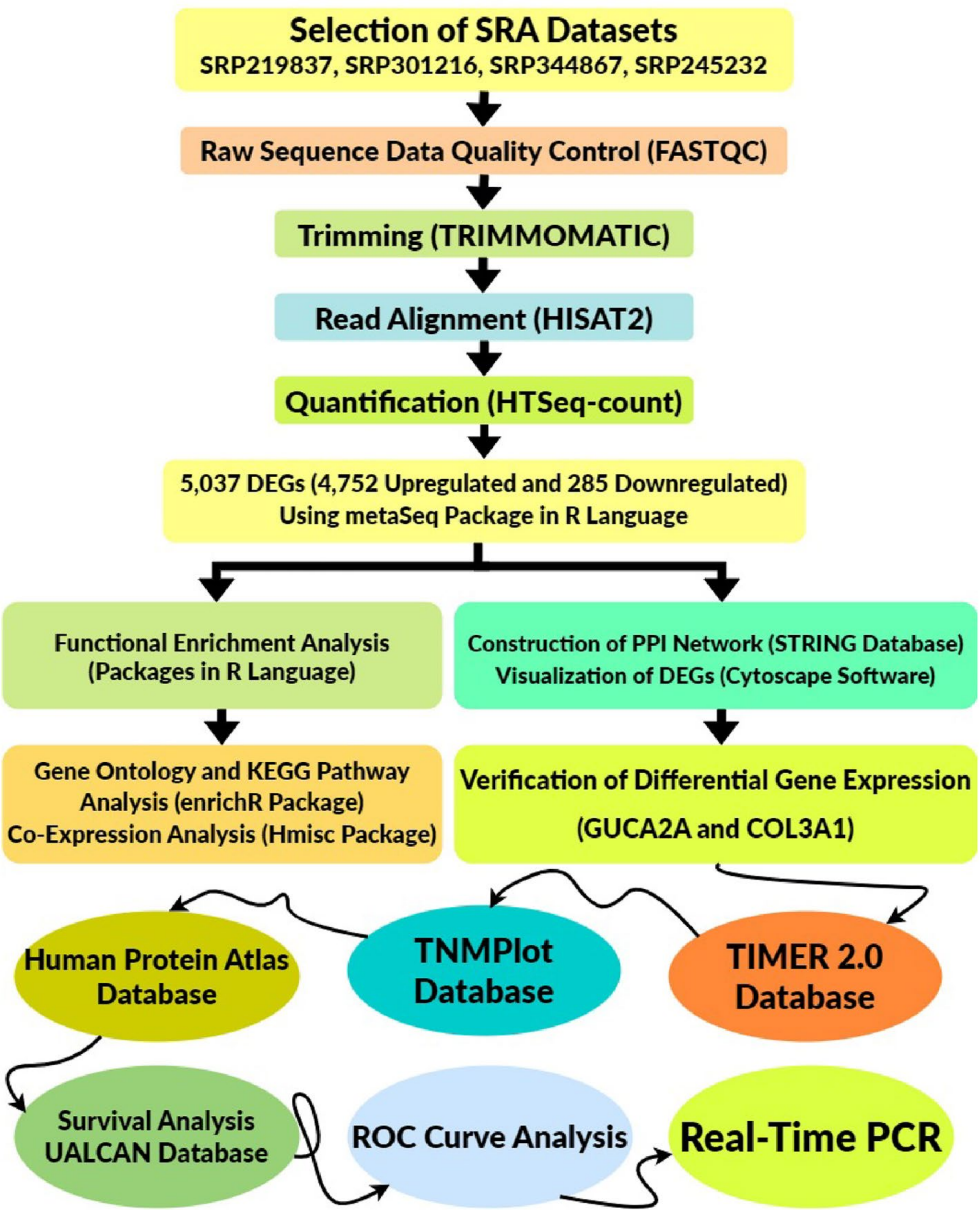
The flowchart in this study. *DEG* differentially expressed genes, *PPI* protein–protein interaction, *GUCA2A* guanylate cyclase activator 2A, *COL3A1* collagen type III alpha 1 chain, *TIMER 2.0* tumor immune estimation resource 2, *ROC Curve* receiver operating characteristic curve.

The general flowchart of data processing and detailed methods are described in Fig. 1. We searched PubMed database and the Gene Expression Omnibus database (GEO, https://www.ncbi.nlm.nih.gov/geo/)^29^ and Sequence Read Archive (SRA, https://www.ncbi.nlm.nih.gov/sra)^30^ to identify RNA-Seq-based CRC expression profiling research. The key words “colorectal cancer, gene expression, RNA-Seq and genetics” and their combinations were searched. Experimentally Bulk RNA-Seq datasets related to gene expression levels in healthy and tumor tissues of colorectal cancer patients were included. RNA-Seq datasets related to research on experimental animals including mice and rats and research related to treating different cell lines with antibodies and different drugs and systematic review articles were not included.

### Information of RNA-Seq data sets

We downloaded four original expression RNA-Seq datasets: SRP219837, SRP301216, SRP344867 and SRP245232 from the SRA database (Available online: https://www.ncbi.nlm.nih.gov/sra) and raw count from the Cancer Genome Atlas (TCGA) (Available online: https://portal.gdc.cancer.gov/). These datasets and counts provided 60 CRC tissues and 60 normal tissues. The SRP219837 dataset included 5 colorectal tumor tissues and 5 adjacent normal tissues^31^. The SRP301216 dataset included 5 CRC tissues and 5 normal colon tissues^32^. The SRP344867 dataset included 5 colon cancer tissues and 4 adjacent normal tissues^33^. The SRP245232 dataset included 3 colon cancer tissues and 3 normal colon tissues^34^. The TCGA datasets included 42 colorectal cancer tissues and 43 normal tissues. Selected details of the individual studies were summarized in Supplementary Table 1.

### Preprocessing of sequencing reads: quality control, trimming, mapping and counting

FASTQC software (https://www.bioinformatics.babraham.ac.uk/projects/fastqc/) was used to check the read quality of the sequences^35^. TRIMMOMATIC tool (V-0.39) was used to remove and trim reads^36^. The sequencing reads were trimmed with the options (LEADING:20, TRAILING:20, SLIDINGWINDOW:4:25, MINLEN:50). Cleaned RNA-Seq data were mapped to human reference genome hg38 using the HISAT2 (v2.2.1) alignment program^37^. Read counts for gene expression were obtained using the HTSeq software^38^.

### Identification of common DEGs

Differential expression genes was assessed by “meta-Seq”^28^ Package in R software using Fisher Method (NOI-Seq), which is the recommended and most common method to estimate the level of gene expression for integrated RNA-Seq data. Differentially expressed genes were selected based on *p* value < 0.05.

### GO and KEGG pathway enrichment analysis of DEGs

GO analysis is a common method used to determine the distinct biological functions of genes and proteins using data obtained by high-throughput sequencing^39^. The KEGG is a group of databases created to systematically examine gene function and connect genomic data with higher level biological function pathways^40^. Therefore, the GO and KEGG pathways enrichment analysis of DEGs were performed using “enrichR”^41^ package in R software. Adj.pvalue < 0.05 was considered as the criterion for statistical significance.

### PPI network construction

The research of protein–protein interactions (PPIs) can help in deciphering the molecular functions of proteins and revealing the rules for cellular functions as differentiation, growth, metabolism, and apoptosis^42^. The identification of protein-interacting ions in a genome-wide scale is essential for the evaluation of the regulatory mechanisms^43^. STRING (Search Tool for the Retrieval of Interacting Genes), an accessible online tool, was used to evaluate the PPI network of common DEGs^44^. The PPI network complex of the common DEGs was then imported into Cytoscape v3.10.0 (https://cytoscape.org/), which is a free software for visualization of PPI networks^45^.

### Co-expression analysis of 40 common DEGs

The “DESeq2”^46^ package (“cor” function) was used to normalized counts in R software. The “Hmisc”^47^ package (“corplot” function) was used to draw the co-expression matrix of DEGs. *p* value < 0.05 was considered statistically significant.

### GUCA2A and COL3A1 in different types of cancer

We used the TIMER2.0 database (http://timer.cistrome.org/)^48–50^ for investigated the expression of GUCA2A and COL3A1 genes in different types of cancer. RNA-seq data was utilized to confirm our final candidate genes using TNMplot (https://tnmplot.com/)^51^.

### Identification of the protein expression levels in hub genes

Immunohistochemistry images from the Human Protein Atlas (HPA) online database (http://www.proteinatlas.org/) were utilized to distinguish between normal and CRC tumor tissues in order to clarify the differential expression of hub genes at a protein level.

### Survival analysis of hub genes

The UALCAN database was used to conduct the survival analysis using data from the TCGA COAD and READ datasets in order to examine for the prognostic values of GUCA2A and COL3A1 in COAD and READ patients^52^. A p value of < 0.05 was used as the cut-off criterion.

### Statistical analysis

Chi-Square and Fisher’s exact tests were used to analyzing the connection between clinical characteristics, such as age, sex, hemoglobin, tumor size (cm), histology grade, lymphatic invasion, vascular invasion, perineural invasion, TNM staging, family history, alcohol and smoking with each gene expression. *p-*value < 0.05 was regarded as a significant association. Based on sensitivity and specificity, the GraphPad Prism software version 9.0 (GraphPad Software, San Diego, CA, USA) conducted the ROC Cure analysis for the RNA-Seq datasets. The Areas Under Curve (AUC) between 0.7 and 0.8 are considered reasonable in the ROC analysis, 0.8–0.9 are good (which represents a good biomarker) and 0.9–1 revealed a particularly unique biomarker. The significance criteria for this analysis were determined to be a *p-*value of less than 0.05.

### CRC patients

The twenty CRC patients diagnosed with rectal cancer or colon cancer (20 tumor and 20 adjacent normal, 9 men and 11 women; age range 28–76 years) included in this study were from the Imam Khomeini Hospital Cancer Institute, Tehran, Iran. All the CRC patients involved in the study were diagnosed with pathological proof and has not been received chemotherapy or radiotherapy before the surgery. The clinicopathological features of each patient are summarized in Table 1. Each tumor sample was matched with a sample of nearby normal mucosa that had been surgically removed. These tissues were divided into frozen sections, which senior pathologists independently examined. After the procedure, paired samples of normal and cancer were immediately frozen and stored at − 80 °C until RNA extraction. This study was performed in accordance with the Helsinki Declaration and all patients participating in the study provided written informed consent. This study is approved by the research ethics committees of Islamic Azad University-Sari Branch with the following ethic code IR.IAU.SARI.REC.1401.026.

**Table 1:** The clinicopathological features of twenty CRC patients for qRT-PCR validation.

No.	Pathological diagnosis	Anatomic site	Pathological TNM staging	Age	Gender	Tumor size (cm)
A00018-1	Adenocarcinoma	Rectum	T2N1M0IIIA	74	Male	9.00
A00019-3	Adenocarcinoma	Colon	T3N1M0IIIB	70	Male	5.00
A00139-5	Adenocarcinoma	Sigmoid colon	T3N1M0IIIB	67	Female	5.00
A00152-7	Adenocarcinoma	Sigmoid colon	T3N0M0IIA	64	Female	2.00
A00155-9	Adenocarcinoma	Sigmoid colon	T3N0M1IV	70	Male	10.50
A00302-11	Adenocarcinoma	Rectosigmoid	T2N0M0I	35	Male	6.00
A00304-13	Adenocarcinoma	Colon	T1N2M1IV	64	Female	10.00
A00314-15	Adenocarcinoma	Sigmoid colon	T3N0M0IIA	54	Female	5.00
A00334-17	Adenocarcinoma	Colon	T3N0M0IIA	55	Male	8.00
A00463-19	Adenocarcinoma	Colon	T3N1M0IIIB	45	Female	4.00
A00469-21	Adenocarcinoma	Colon	T3N1M1IV	37	Female	5.50
A00504-23	Adenocarcinoma	Rectum	T3N1M0IIIB	64	Female	2.50
A00540-25	Adenocarcinoma	Colon	T4N1M0IIIB	64	Female	6.00
A00585-27	Adenocarcinoma	Rectosigmoid	T3N1M0IIIB	60	Female	2.50
A00684-29	Adenocarcinoma	Colon	T2N0M0I	64	Male	7.00
A00710-31	Adenocarcinoma	Rectosigmoid	T3N0M1IV	53	Male	6.00
A00742-33	Adenocarcinoma	Ascending colon	T3N2M1IV	57	Female	8.50
A00835-35	Adenocarcinoma	Rectum	T3N1M1IV	44	Female	3.00
A00883-37	Adenocarcinoma	Rectosigmoid	T3N2M1IV	28	Male	4.50
A00899-39	Adenocarcinoma	Rectum	T4N0M1IV	76	Male	12.00

### RNA extraction, cDNA synthesis and qRT-PCR

The total RNA from each sample was extracted using Trizol Reagent (YTzole, Yekta Tajhiz Co. Tehran, Iran) according to the manufacturer’s protocol. The NanoDrop™ 2000/2000c Spectrophotometers (Termo Fisher Scientific, USA) were used for calculating absorbance and concentration with the goal to evaluate RNA. The 260/230 nm and 260/280 nm absorption ratio were evaluated. Both the ratios of 1.8 to 2.2 and 1.7 to 1.9 were regarded as appropriate values. We utilized OLIGO Primer Analysis Software Version 7 (Molecular Biology Insights, Inc., Cascade, CO, USA)^53^ and Primer3Plus^54^ to design primers for SYBR-Green experiments using template sequences and we adopted MIC Real Time PCR Cycler (Bio Molecular Systems, Queensland, Australia). The primers for the qRT-PCR are listed in Table 2 and synthesized by metabion international AG Company (Planegg, Germany). For each replicate, complementary DNA (cDNA) was synthesized from 1 to 5 μg RNA using cDNA Synthesis Kit (ROJE Technologies Co. Tehran, Iran). The qRT-PCR reaction comprised 5 μl of YTA SYBR Green qPCR Master Mix 2X (Yekta Tajhiz Co. Tehran, Iran), 500 ng of diluted cDNA and 1 μM of each primer contributing a total volume of 10 μl. Reactions were conducted in duplicate to insure consistent technical replication and then run in 48-well MIC PCR under the following conditions: 95 °C for 20 min, 40 cycles of 95 °C for 15 s and 63.2 °C for 15 s, and 72 °C for 20 s. Melting curves (72–95 °C) were derived for every reaction to insure a single product. Relative gene expression was evaluated with Bio Molecular Systems software version 2.12 (Queensland, Australia) and using human GAPDH gene as the endogenous control for RNA load and gene expression in analysis. The qRT-PCR results were analyzed using GraphPad Prism Software version 9.0 (GraphPad Software, San Diego, CA, USA). Next, Unpaired Student’s t-test was used to determine the statistical significance of the difference between normally distributed variables, and a p-value of 0.05 or less was considered as statistically significant.

**Table 2:** Details of primers used in Real-time PCR.

Gene	Sequence	PCR product	RefSeq ID	Ta (°C)
GUCA2A	F: TGTGGTTCCCATCCTCTGTAG	143	NM_033553.3	61
	R: CAGCGTAGGCACAGATTTCAC			
COL3A1	F: TTCTCGCTCTGCTTCATCCC	88	NM_000090.4	60
	R: TCCGCATAGGACTGACCAAG			
GAPDH (human)	F: ACAGGGTGGTGGACCTCAT	175	NM_001256799.3	60
	R: AGGGGTCTACATGGCAACTG			

## Results

### Preprocessing of sequencing reads

FASTQC was used to evaluate the raw sequenced read quality from RNA-seq studies and it found very high quality. Regardless of the raw data quality, all samples underwent standard data cleaning to make sure that no base was called with a phred quality lower than 20. Summary of RNA-Seq analysis results present in Supplementary Table 2.

### Identification of common DEGs in CRC

There were 60 CRC tissues and 60 normal colorectal tissues samples used in this study. After integrated analysis, with a *p* value < 0.05, 5037 DEGs (4752 upregulated, 285 downregulated) were found to show altered expression in samples of CRC compared with normal tissues. Furthermore, a list of the top 40 most significantly differential expression genes was presented in Table 3. GUCA2A plays an important role in the transformation of polyps into colorectal cancer tissue^55^, COL3A1 associated with colorectal cancer lymph node metastasis^56^ and based on our in silico analysis, these two genes were selected as hub genes and finally selected for experimental validation. Full list of DEGs between cancer tissues and normal tissues were shown in Supplementary Table 3.

**Table 3:** The top 40 most significantly DEGs.

Up regulated			Down regulated		
Gene symbol	Official full name	p value	Gene symbol	Official full name	p value
KCNQ1OT1	KCNQ1 opposite strand/antisense transcript 1	2.21E−06	GUCA2A	Guanylate cyclase activator 2A	0.0003
KRT6A	Keratin 6A	6.80E−06	PYY	Peptide YY	0.0003
KRT6B	Keratin 6B	9.37E−06	AQP8	Aquaporin 8	0.0005
KRT17	Keratin 17	9.43E−06	GUCA2B	Guanylate cyclase activator 2B	0.001
KRT16	Keratin 16	2.18E−05	ZG16	Zymogen granule protein 16	0.001
COL3A1	Collagen type III alpha 1 chain	2.20E−05	CD177	CD177 molecule	0.001
PLAC4	Placenta enriched 4	2.67E−05	IGHA2	Immunoglobulin heavy constant alpha 2 (A2m marker)	0.002
COL1A1	Collagen type I alpha 1 chain	2.92E−05	CLCA1	Chloride channel accessory 1	0.002
KRT5	Keratin 5	4.02E−05	UGT2B17	UDP glucuronosyltransferase family 2 member B17	0.002
COL1A2	Collagen type I alpha 2 chain	4.60E−05	APOB	Apolipoprotein B	0.002
MAGEB17	MAGE family member B17	4.62E−05	CLCA4	Chloride channel accessory 4	0.002
CXCL8	C-X-C motif chemokine ligand 8	4.69E−05	SYNM	Synemin	0.003
RMRP	RNA component of mitochondrial RNA processing endoribonuclease	5.70E−05	MT1M	Metallothionein 1M	0.003
ATP6V1C2	ATPase H + transporting V1 subunit C2	6.82E−05	SLC6A19	Solute carrier family 6 member 19	0.003
CEACAM6	CEA cell adhesion molecule 6	7.38E−05	MT1G	Metallothionein 1G	0.003
SPARC	Secreted protein acidic and cysteine rich	0.0001	PADI2	Peptidyl arginine deiminase 2	0.004
HSP90AB1	Heat shock protein 90 alpha family class B member 1	0.0001	APOA4	Apolipoprotein A4	0.004
SLCO4A1-AS1	SLCO4A1 antisense RNA 1	0.0001	OTOP2	Otopetrin 2	0.004
ACTG1	Actin gamma 1	0.0001	ANPEP	Alanyl aminopeptidase, membrane	0.005
KRT6C	Keratin 6C	0.0001	ADH1B	Alcohol dehydrogenase 1B (class I), beta polypeptide	0.005

### GO and KEGG pathway enrichment analysis of DEGs in CRC

#### GO analysis for downregulated genes

The GO analysis revealed that the highest rate of down-regulated DEGs were involved in (1) cellular response to zinc ion (GO:0071294, padj:2.21E−06), cellular zinc ion homeostasis (GO:0006882, padj:2.21E−06), muscle contraction (GO:0006936, padj:2.21E−06), cellular response to copper ion (GO:0071280, padj:2.21E−06), zinc ion homeostasis (GO:0055069, padj:2.52E−06), found in the BP category; (2) brush border membrane (GO:0031526, padj:6.52E−06), actin cytoskeleton (GO:0015629, padj:0.0002), cell projection membrane (GO:0031253, padj:0.0002), Chylomicron (GO:0042627, padj:0.0002), Sarcolemma (GO:0042383, padj:0.0002), found in the CC category; (3) transition metal ion binding (GO:0046914, padj:1.26E−06), metal ion binding (GO:0046872, padj:1.26E−06), zinc ion binding (GO:0008270, padj:1.00E−05), actin binding (GO:0003779, padj:2.33E−05), calcium ion binding (GO:0005509, padj:0.0007), found in the MF category (Supplementary Fig. S1, Table 4). Complete lists of all the GO BP, GO CC, and GO MF are presented in Supplementary Table 4.

**Table 4:** Gene ontology analysis results for down-regulated genes.

Category	GO ID	GO term	Count	p value	padj
Biological process	GO:0071294	Cellular response to zinc ion	7	3.08E−09	2.21E−06
	GO:0006882	Cellular zinc ion homeostasis	8	3.74E−09	2.21E−06
	GO:0006936	Muscle contraction	14	4.66E−09	2.21E−06
	GO:0071280	Cellular response to copper ion	7	4.82E−09	2.21E−06
	GO:0055069	Zinc ion homeostasis	8	6.87E−09	2.52E−06
	GO:0046688	Response to copper ion	7	2.23E−08	6.84E−06
	GO:0072503	Cellular divalent inorganic cation homeostasis	12	6.25E−08	1.64E−05
	GO:0071276	Cellular response to cadmium ion	7	7.71E−08	1.77E−05
	GO:0046916	Cellular transition metal ion homeostasis	11	1.22E−07	2.06E−05
	GO:0046686	response to cadmium ion	7	1.32E−07	2.06E−05
Cellular component	GO:0031526	Brush border membrane	8	4.15E−08	6.52E−06
	GO:0015629	Actin cytoskeleton	17	3.19E−06	0.0002
	GO:0031253	Cell projection membrane	9	6.59E−06	0.0002
	GO:0042627	Chylomicron	4	7.92E−06	0.0002
	GO:0042383	Sarcolemma	7	8.56E−06	0.0002
	GO:0062023	Collagen-containing extracellular matrix	17	3.50E−05	0.0008
	GO:0005887	Integral component of plasma membrane	40	4.68E−05	0.0008
	GO:0034385	TriglyceridE−rich plasma lipoprotein particle	4	4.86E−05	0.0008
	GO:0034361	very-low-density lipoprotein particle	4	4.86E−05	0.0008
	GO:0005856	Cytoskeleton	22	5.37E−05	0.0008
Molecular function	GO:0046914	Transition metal ion binding	25	6.17E−09	1.26E−06
	GO:0046872	Metal ion binding	27	7.05E−09	1.26E−06
	GO:0008270	Zinc ion binding	20	8.42E−08	1.00E−05
	GO:0003779	Actin binding	14	2.60E−07	2.33E−05
	GO:0005509	Calcium ion binding	17	1.14E−05	0.0007
	GO:0004089	Carbonate dehydratase activity	4	1.23E−05	0.0007
	GO:0004177	Aminopeptidase activity	5	3.54E−05	0.001
	GO:0008238	Exopeptidase activity	6	0.0001	0.004
	GO:0120020	Cholesterol transfer activity	4	0.0001	0.004
	GO:0120015	Sterol transfer activity	4	0.0001	0.004

#### GO analysis for upregulated genes

The GO analysis revealed that the highest rate of up-regulated DEGs are enriched in (1) RNA export from nucleus (GO:0006405, padj:9.36E−11), mRNA export from nucleus (GO:0006406, padj:9.36E−11), mRNA transport (GO:0051028, padj:1.60E−10), mRNA-containing ribonucleoprotein complex export from nucleus (GO:0071427, padj:1.60E−10), mitotic spindle organization (GO:0007052, padj:1.45E−09), found in the BP category; (2) Nucleolus (GO:0005730, padj:3.52E−10), nuclear lumen (GO:0031981, padj:4.15E−10), Chromosome (GO:0005694, padj:2.08E−09), intracellular non-membrane-bounded organelle (GO:0043232, padj:8.52E−08), nuclear chromosome (GO:0000228, padj:2.85E−05), found in the CC category; (3) RNA binding (GO:0003723, padj:8.68E−20), DNA replication origin binding (GO:0003688, padj:0.002), single-stranded DNA binding (GO:0003697, padj:0.01), CXCR chemokine receptor binding (GO:0045236, padj:0.01), mRNA binding (GO:0003729, padj:0.01), found in the MF category (Supplementary Fig. S2, Table 5). Complete lists of all the GO BP, GO CC, and GO MF are presented in Supplementary Table 5.

**Table 5:** Gene ontology analysis results for up-regulated genes.

Category	GO ID	GO term	Count	p value	padj
Biological process	GO:0006405	RNA export from nucleus	62	1.90E−14	9.36E−11
	GO:0006406	mRNA export from nucleus	62	3.48E−14	9.36E−11
	GO:0051028	mRNA transport	60	1.17E−13	1.60E−10
	GO:0071427	mRNA-containing ribonucleoprotein complex export from nucleus	58	1.19E−13	1.60E−10
	GO:0007052	Mitotic spindle organization	78	1.35E−12	1.45E−09
	GO:1902850	Microtubule cytoskeleton organization involved in mitosis	67	2.36E−12	2.12E−09
	GO:0051031	tRNA transport	29	3.60E−12	2.77E−09
	GO:0000398	mRNA splicing, via spliceosome	116	6.69E−12	4.50E−09
	GO:0006409	tRNA export from nucleus	27	1.16E−11	5.74E−09
	GO:0071431	tRNA-containing ribonucleoprotein complex export from nucleus	27	1.16E−11	5.74E−09
Cellular component	GO:0005730	Nucleolus	258	8.13E−13	3.52E−10
	GO:0031981	Nuclear lumen	260	1.91E−12	4.15E−10
	GO:0005694	Chromosome	77	1.43E−11	2.08E−09
	GO:0043232	Intracellular non-membrane-bounded organelle	363	7.85E−10	8.52E−08
	GO:0000228	Nuclear chromosome	41	3.28E−07	2.85E−05
	GO:0001533	Cornified envelope	24	6.13E−06	0.0004
	GO:0071162	CMG complex	9	1.88E−05	0.001
	GO:0000307	Cyclin-dependent protein kinase holoenzyme complex	18	2.36E−05	0.001
	GO:0000793	Condensed chromosome	27	2.48E−05	0.001
	GO:0005685	U1 snRNP	12	0.0001	0.005
Molecular function	GO:0003723	RNA binding	491	7.93E−23	8.68E−20
	GO:0,003688	DNA replication origin binding	16	4.26E−06	0.002
	GO:0003697	Single-stranded DNA binding	41	4.21E−05	0.01
	GO:0045236	CXCR chemokine receptor binding	12	5.78E−05	0.01
	GO:0003729	mRNA binding	90	7.45E−05	0.01
	GO:0016423	tRNA (guanine) methyltransferase activity	10	7.87E−05	0.01
	GO:0030515	snoRNA binding	17	0.0001	0.01
	GO:0003730	mRNA 3ʹ-UTR binding	35	0.0002	0.03
	GO:0017116	Single-stranded DNA helicase activity	12	0.0002	0.03
	GO:0005685	U1 snRNP	12	0.0001	0.005

#### KEGG pathway enrichment analysis of DEGs

Downregulated DEGs were particularly enriched in mineral absorption, fat digestion and absorption, PPAR signaling pathway, Pancreatic secretion and Nitrogen metabolism, whilst the Cell cycle, Spliceosome, RNA transport, DNA replication and Systemic lupus erythematosus were identified as the most represented pathways for the upregulated DEGs (Supplementary Fig. S3, Table 6). Complete lists of all KEGG pathway terms are presented in Supplementary Table 6.

**Table 6:** KEGG pathway analysis results for DEGs.

DEGs	Term	Count	p value	padj
Down regulated	Mineral absorption	13	2.16E−12	4.08E−10
	Fat digestion and absorption	9	7.87E−09	7.44E−07
	PPAR signaling pathway	10	9.48E−08	5.97E−06
	Pancreatic secretion	10	1.98E−06	9.35E−05
	Nitrogen metabolism	5	3.05E−06	0.0001
	Bile secretion	9	5.50E−06	0.0001
	Proximal tubule bicarbonate reclamation	5	1.54E−05	0.0004
	Aldosterone-regulated sodium reabsorption	5	0.0001	0.004
	Renin-angiotensin system	4	0.0002	0.005
	Sulfur metabolism	3	0.0003	0.005
Up regulated	Cell cycle	67	3.30E−13	1.04E−10
	Spliceosome	69	1.88E−09	2.97E−07
	RNA transport	79	1.21E−08	1.28E−06
	DNA replication	21	9.18E−06	0.0007
	Systemic lupus erythematosus	54	1.92E−05	0.001
	mRNA surveillance pathway	41	5.59E−05	0.002
	IL-17 signaling pathway	39	0.0001	0.004
	Neutrophil extracellular trap formation	64	0.001	0.04
	Proteasome	20	0.002	0.08
	Ribosome biogenesis in eukaryotes	39	0.002	0.08

### Protein–protein network complex and hub genes analysis

**Figure 2:**
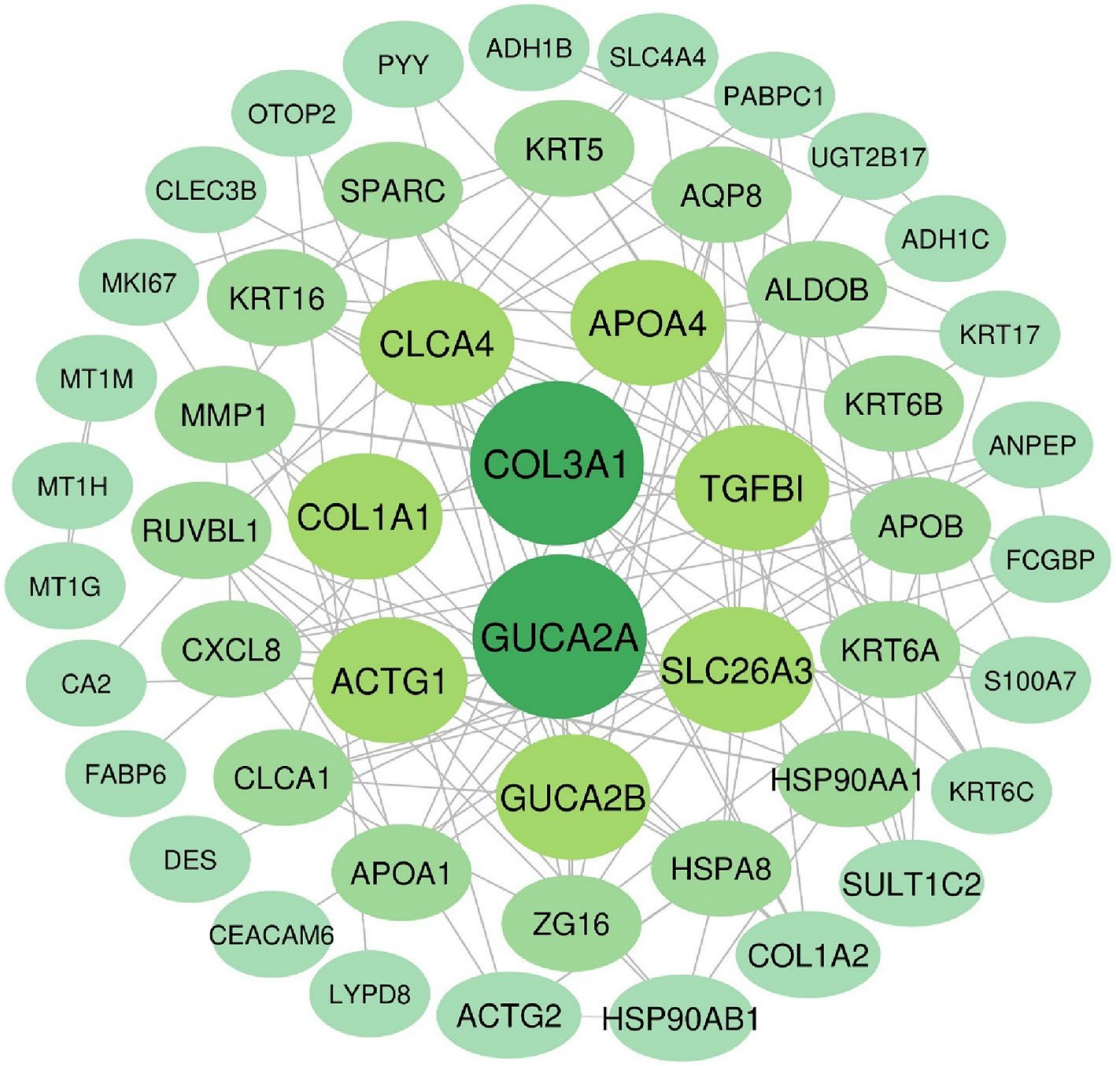
The PPI network of the 80 dysregulated differentially expressed genes (DEGs). 51 out of the 80 DEGs were contained in the PPI network complex. The PPI network of genes from the outside to the inside, according to degree from low to high.

Protein–protein interaction of 80 most significantly DEGs were established by using STRING (https://string-db.org/) and cytoscape software, which included 68 nodes and 121 edges. The 51 genes included 23 up-regulated and 28 down-regulated genes, whilst the remaining 29 genes were not found in a PPI network complex. The significant hub proteins contained GUCA2A and COL3A1 (degree = 9), APOA4, SLC26A3, TGFBI, CLCA4, ACTG1, GUCA2B and COL1A1 (degree = 8), CXCL8, HSP90AA1, HSPA8, RUVBL1, ALDOB, APOB, APOA1, SPARC, AQP8, CLCA1, MMP1, KRT16 and ZG16 (degree = 7), KRT5, KRT6B and KRT6A (degree = 6), HSP90AB1, ACTG2, SULT1C2 and COL1A2 (degree = 5) (Fig. 2).

### Co-expression matrix analysis of 40 common DEGs

**Figure 3:**
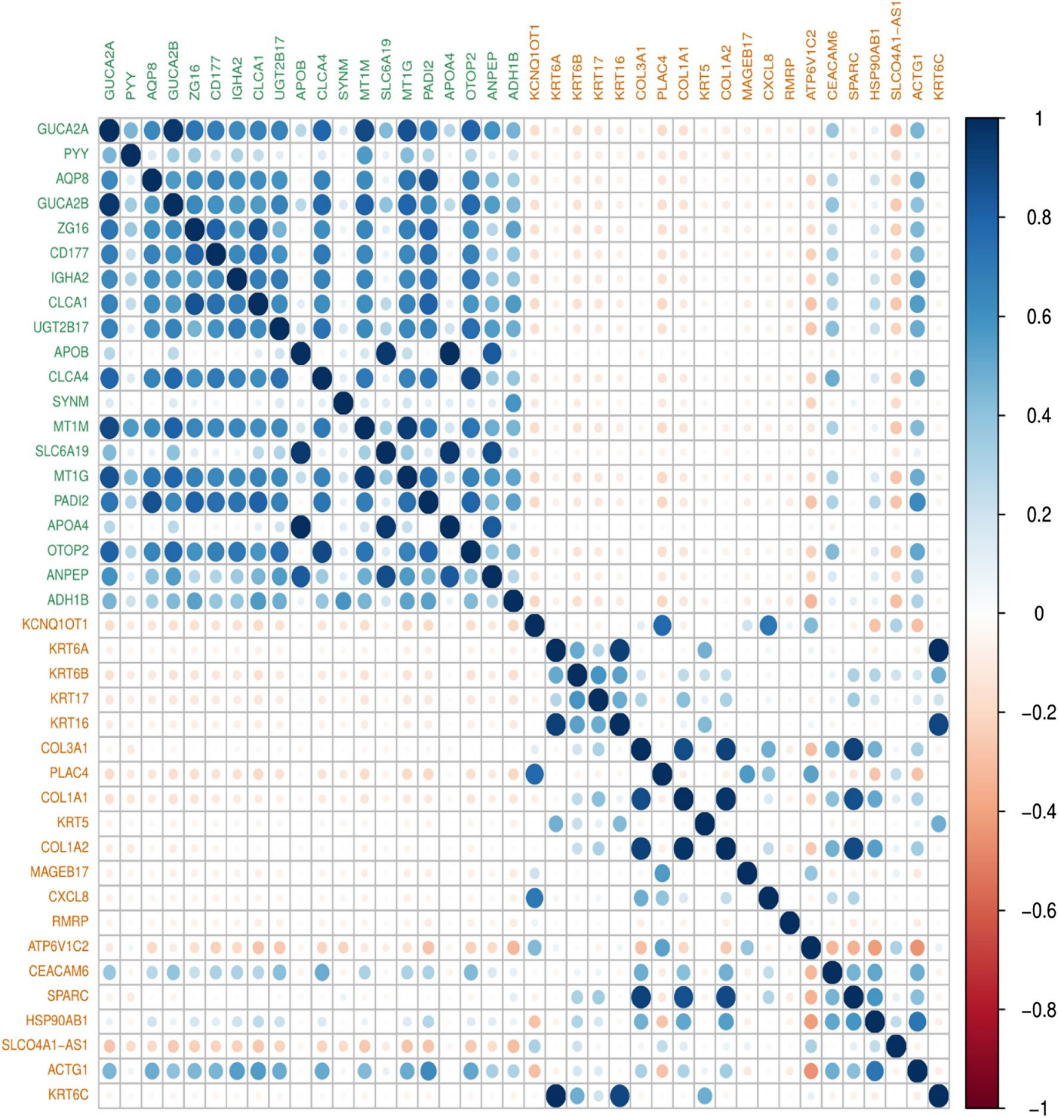
Correlation matrix plot showing the correlation coefficient between 40 common DEGs. The color scale on the right indicates the strengths of the correlations (blue for positive correlation, red for negative correlation), (green color for down-regulated genes and orange color for up-regulated genes).

Within the down-regulated group, one set of genes (GUCA2A, PYY, AQP8, GUCA2B, ZG16, CD177, IGHA2, CLCA1, UGT2B17, APOB, CLCA4, SYNM, MT1M, SLC6A19, MT1G, PADI2, APOA4, OTOP2, ANPEP and ADH1B) showed positive correlation within the group, and negative correlation with the other set of genes (KCNQ1OT1, KRT6A, KRT6B, KRT17, KRT16, COL3A1, PLAC4, COL1A1, KRT5, COL1A2, MAGEB17, CXCL8, RMRP, ATP6V1C2, CEACAM6, SPARC, HSP90AB1, SLCO4A1-AS1, ACTG1, KRT6C) in the up-regulated group. For the GUCA2A, AQP8, GUCA2B, ZG16, CD177, IGHA2, CLCA1, UGT2B17, CLCA4, MT1M, MT1G, PADI2, OTOP2 showed the strongest positive correlation within the group, and weaker or negative correlation with the second set of genes (KCNQ1OT1, KRT6A, KRT6B, KRT17, KRT16, PLAC4, COL1A1, MAGEB17, CXCL8, RMRP, SLCO4A1-AS1) in the up-regulated group (Fig. 3). Numerical value and p-value for co-expression matrix of 40 DEGs present in Supplementary Table 7.

### GUCA2A and COL3A1 expression in human colorectal cancer

**Figure 4:**
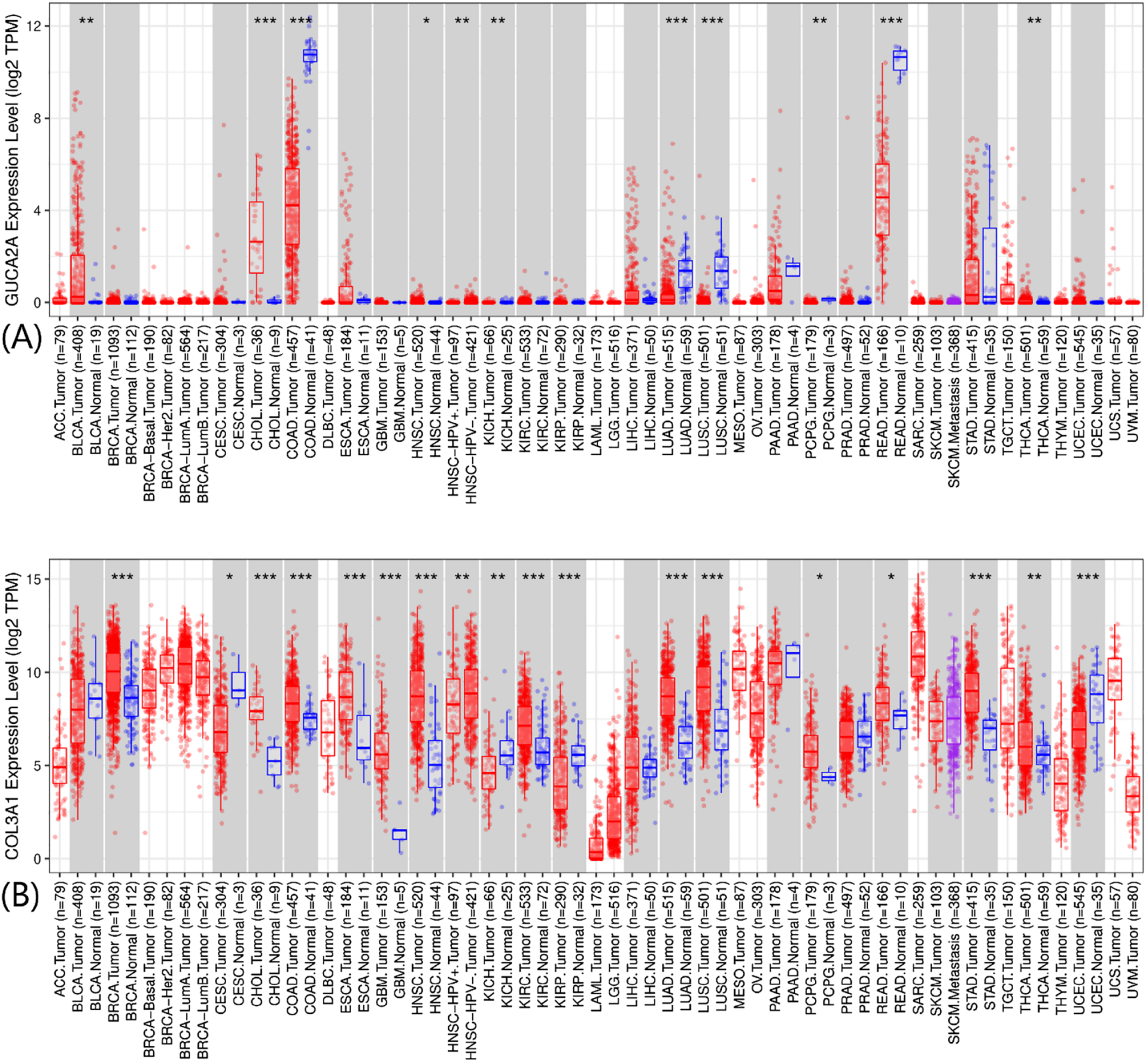
Human GUCA2A and COL3A1 expression levels in different tumor types from TCGA database were determined by TIMER 2.0. (**A**) Comparative expression of GUCA2A. (**B**) Comparative expression of COL3A1 (*p < 0.05; **p < 0.01; ***p < 0.001).

**Figure 5:**
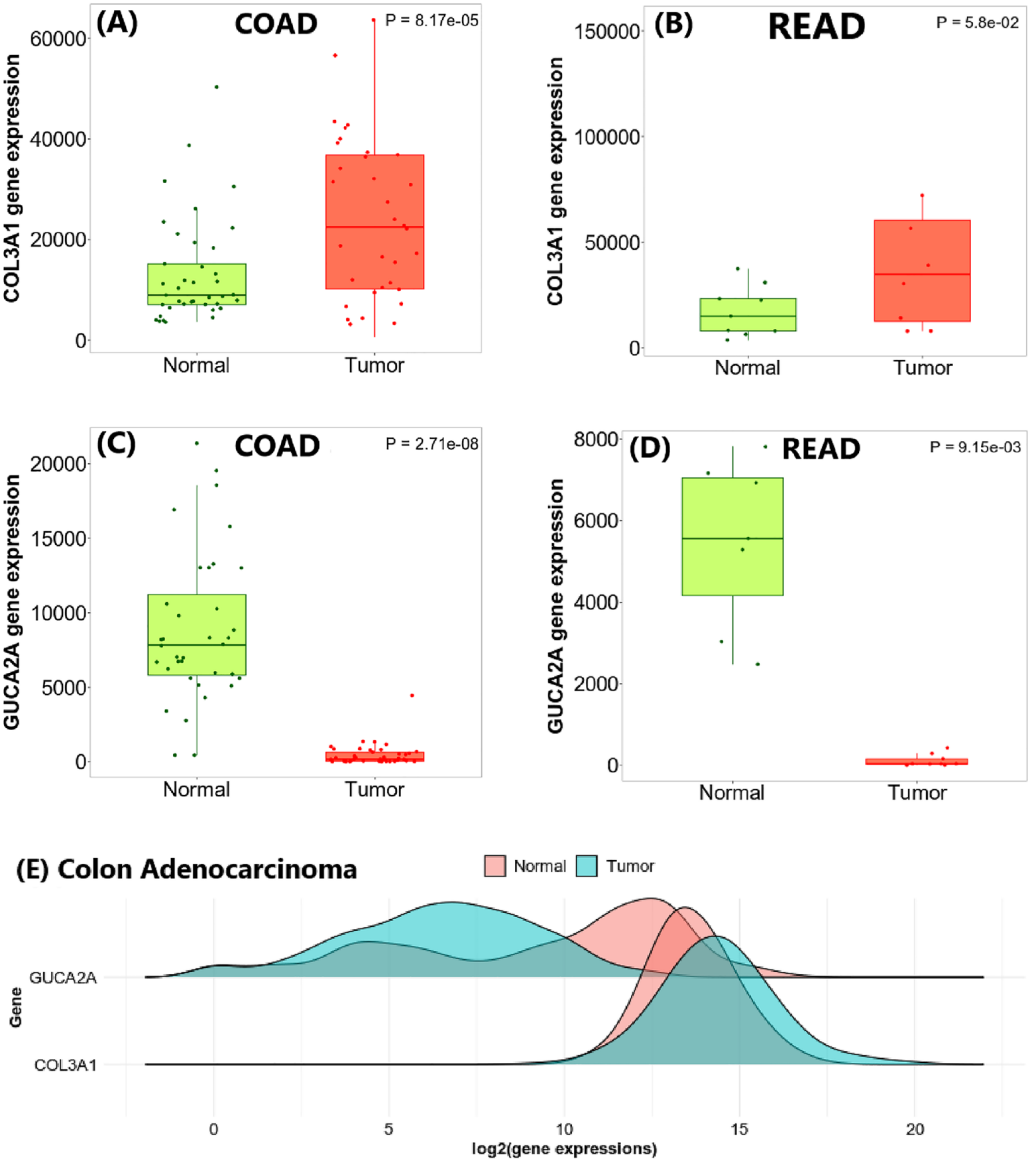
TNM plot of candidate genes which are evaluated in RNA-seq technique. Box plot for (**A**) COL3A1 in colon adenocarcinoma, (**B**) COL3A1 in rectum adenocarcinoma, (**C**) GUCA2A in colon adenocarcinoma, (**D**) GUCA2A in rectum adenocarcinoma, (**E**) density plot for GUCA2A and COL3A1 in colon adenocarcinoma.

We used the TIMER2.0. tool to screen for GUCA2A and COL3A1 expression in multiple cancer types and found that GUCA2A was significantly downregulated in 11 cancer type (Fig. 4A) and COL3A1 was significantly upregulated in 18 cancer type (Fig. 4B), especially in colon and rectal adenocarcinoma. Additionally, in the RNA-seq method, TNMplot and DensityPlot demonstrated a similar different expression pattern for candidate genes in colon and rectum adenocarcinoma (Fig. 5). These finding suggest that GUCA2A and COL3A1 may be essential in the initiation and progression of CRC. *p* value for GUCA2A and COL3A1 expression presented in supplementary table 8.

### Immunohistochemistry validation using human protein atlas database

**Figure 6:**
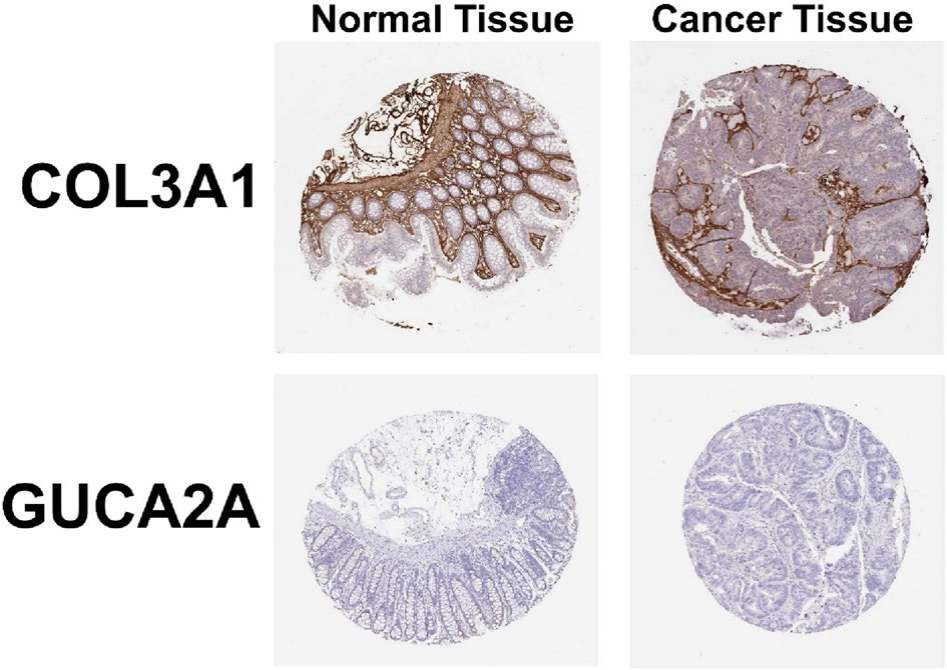
Protein expression of COL3A1 and GUCA2A genes with immunochemistry assay in normal and cancer tissues using HPA database.

Using immunohistochemical images from the Human Protein Atlas database, the hub genes’ protein expressions were confirmed. GUCA2A were low expressed in both normal and tumor tissues and COL3A1 was positively expressed in both normal and tumor tissues, but significantly stronger in certain tumor tissues (Fig. 6).

### Evaluation of the diagnostic performance of GUCA2A and COL3A1 in CRC

**Figure 7:**
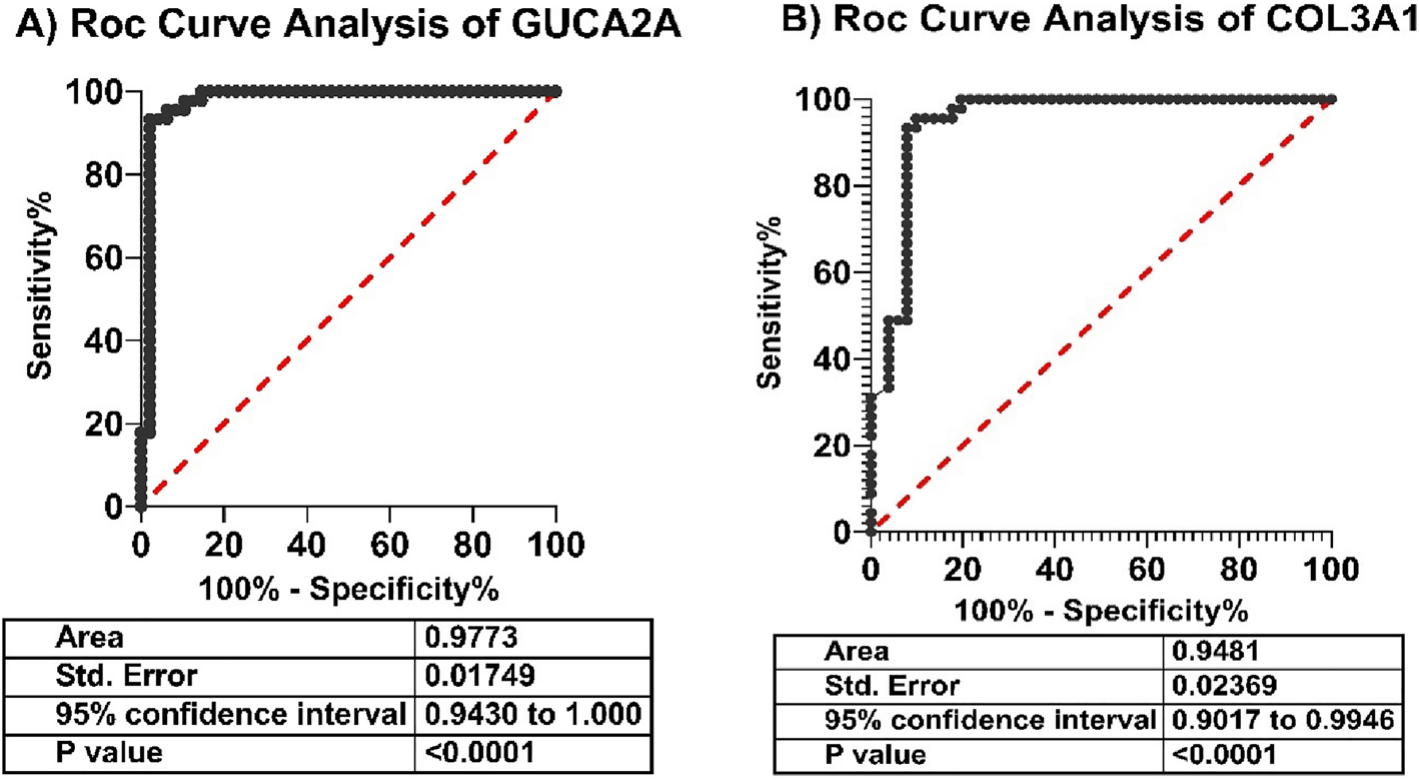
The results of receiver operating characteristic (ROC) curve analysis for the diagnostic value of genes including (**A**) GUCA2A and (**B**) COL3A1, obtained from TCGA data.

**Figure 8:**
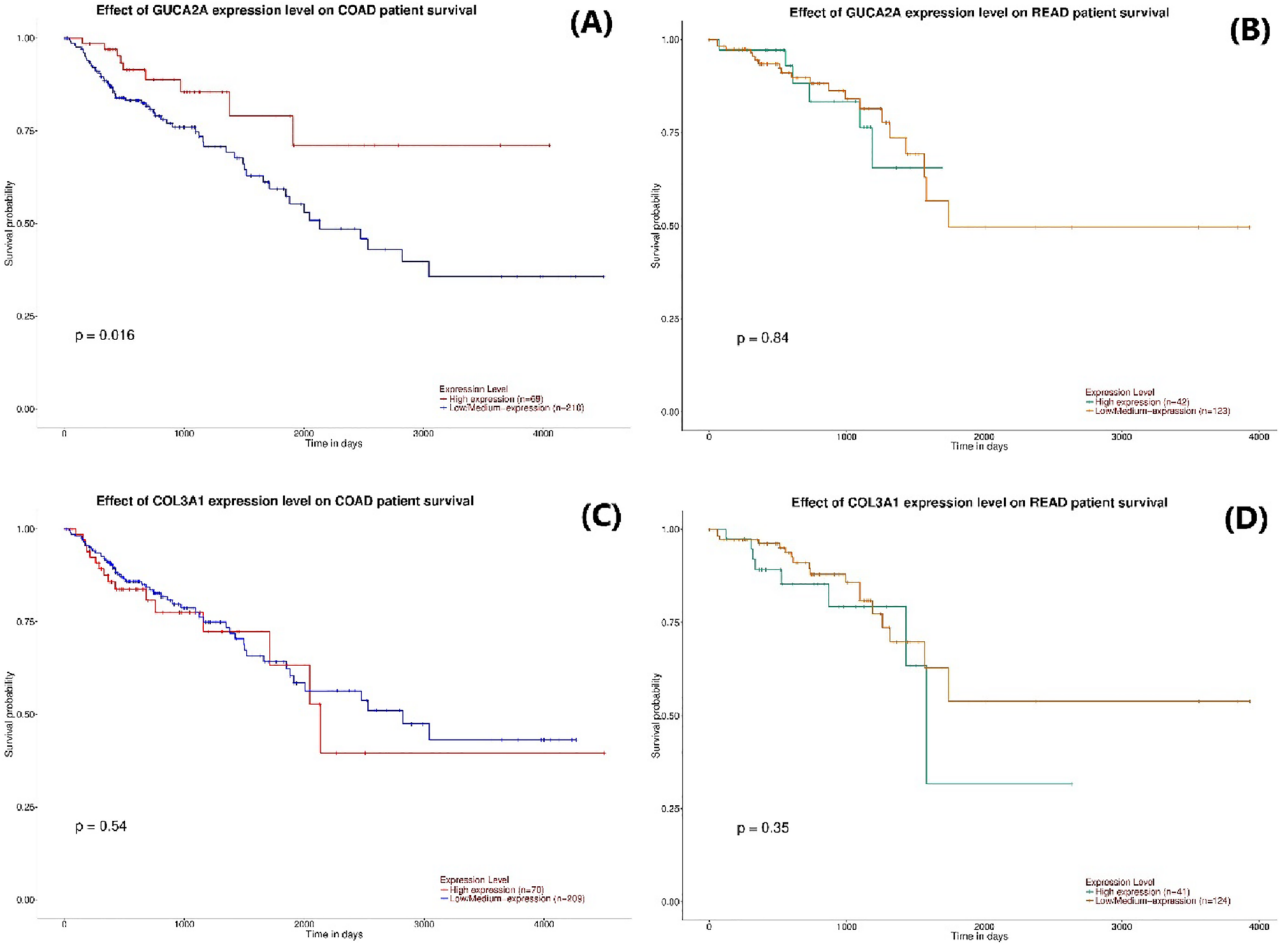
Survival analysis of GUCA2A in (**A**) colon adenocarcinoma, (**B**) rectum adenocarcinoma and COL3A1 in (**C**) colon adenocarcinoma, (**D**) rectum adenocarcinoma using UALCAN database.

We carried out ROC curves and survival analysis for evaluating the efficacy of the identified genes for the diagnosis of colorectal cancer tumor and survival rate of patients. The potential of GUCA2A and COL3A1 expression level as a diagnostic biological parameter to distinguish CRC patients from healthy controls was demonstrated by ROC curve assessment, which was utilized to determine the sensitivity and specificity of GUCA2A expression (AUC 0.9773, 95% CI 0.9430 to 1.000, *p-*value < 0.0001) (Fig. 7A) and COL3A1 expression (AUC 0.9481, 95% CI 0.9017 to 0.9946, *p-*value < 0.0001) (Fig. 7B) for CRC diagnosis. This demonstrates that the expression of COL3A1 and GUCA2A can be beneficial as a tumor biomarker. Survival analysis by UALCAN database revealed that low expression of GUCA2A was significantly associated with lower survival rates of colon adenocarcinoma patients (Fig. 8A) and low expression of GUCA2A is not significantly correlated with rectal adenocarcinoma’s poor prognosis (Fig. 8B). Also, the survival analysis revealed that the high expression of COL3A1 has a not-significant relation with the low survival rate of colon adenocarcinoma (Fig. 8C) and rectal adenocarcinoma patients (Fig. 8D).

### The association between COL3A1 and GUCA2A expression with histopathological characteristics of patients

We investigated the association between each gene expression and the histopathological characteristics of the patients such as age, sex, hemoglobin rate, tumor size (cm), histology grade, lymphatic invasion, vascular invasion, perineural invasion, TNM staging, family history, alcohol and smoking. The listed characteristics of patients were not significantly associated with GUCA2A and COL3A1 gene expression (Table 7) (p-value > 0.05).

**Table 7:** Correlation of genes expression with clinicopathological characteristics in CRC tumors.

Characteristic	Number	*p*-value			
		COL3A1 Colon	COL3A1 Rectum	GUCA2A Colon	GUCA2A Rectum
Age		0.43	1.0	1.0	0.14
< 50	5				
> 50	15				
Sex		0.87	0.84	0.91	0.67
Male	9				
Female	11				
Hemoglobin		1.0	0.88	1.0	1.0
< 10	3				
> 10	17				
Tumor size (cm)		1.0	0.47	1.0	0.74
< 5	6				
≥ 5	14				
Histology grade		0.54	0.87	0.54	1.0
Grade I	14				
Grade II	6				
Lymphatic invasion		0.56	0.97	0.47	0.69
Yes	9				
No	11				
Vascular invasion		0.24	1.0	1.0	1.0
Yes	10				
No	10				
Perineural invasion		1.0	1.0	1.0	1.0
Yes	12				
No	8				
TNM staging		0.54	0.41	0.54	0.41
Stage I	2				
Stage IIA	3				
Stage IIIA	1				
Stage IIIB	6				
Stage IV	8				
Family history		0.06	0.41	1.0	1.0
Yes	7				
No	13				
Alcohol		1.0	1.0	1.0	1.0
Non-drinker	20				
Drinker	0				
Smoking		0.18	1.0	1.0	1.0
Non-smoker	17				
Smoker	3				

### GUCA2A and COL3A1 expression patterns in RNA-Seq data, colon and rectal cancer tissues

**Figure 9:**
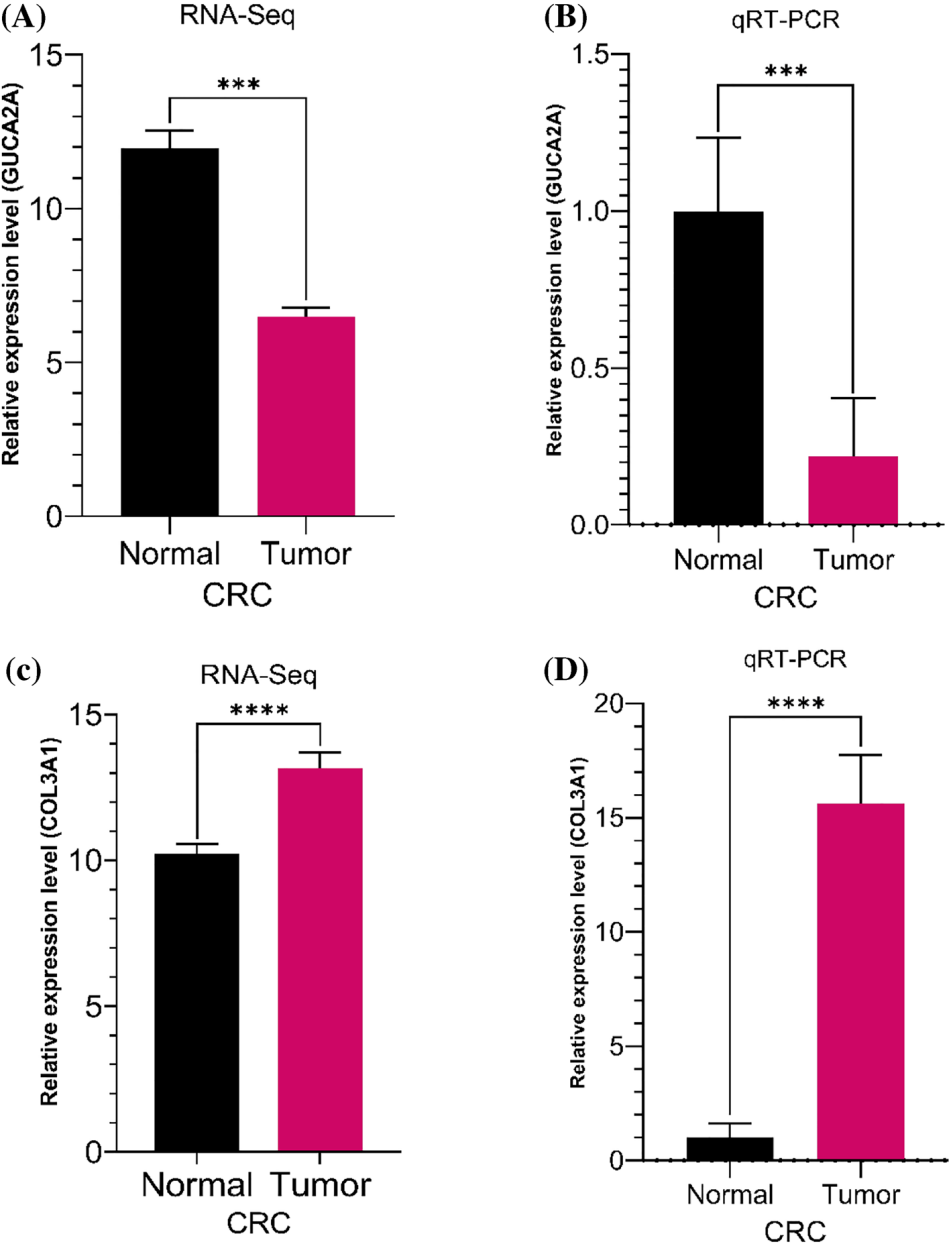
Expression levels of GUCA2A and COL3A1 from RNA-Seq (read counts) and qRT-PCR (2^−△△ct^).

**Figure 10:**
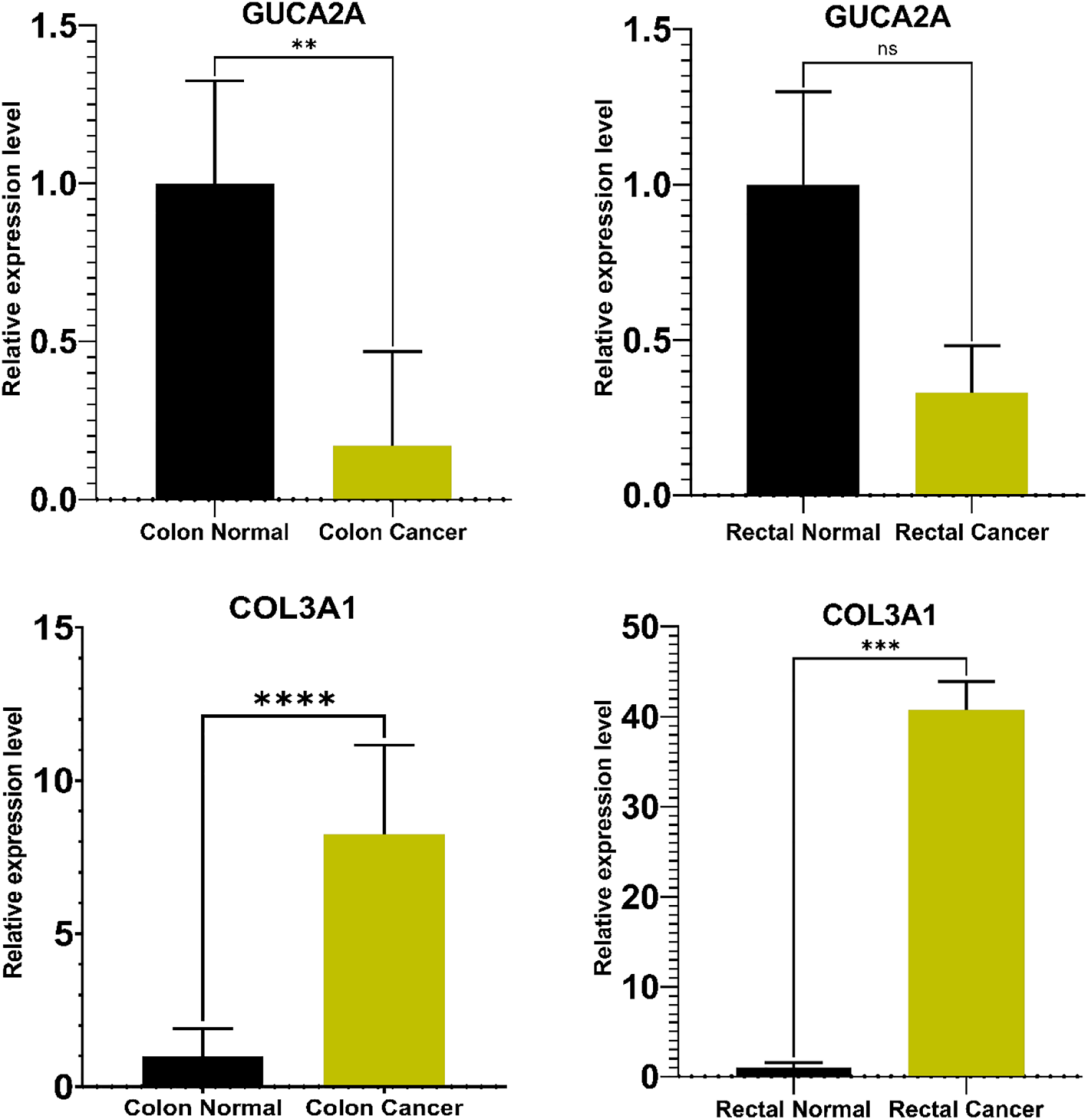
Quantitative real-time polymerase chain reaction (qRT-PCR) analysis data for GUCA2A and COL3A1 are presented in colon cancer and rectal cancer. Two technical replicates were performed for each sample. The height of each box represents the mean average of sample specific 2^−△△ct^ values, while associated error bars denote the S.E.M. fold changes are show in parentheses.

We performed qRT-PCR for GUCA2A and COL3A1 in colon and rectal cancer. GUCA2A were significantly downregulated in tumor tissues compared with normal tissues (− 0.41-fold, *p-*value: 0.0007) (Fig. 9B) and COL3A1 were significantly upregulated in tumor tissues comparison with healthy tissues (7.18-fold, p-value: 0.0001) (Fig. 9D). Our results demonstrated that GUCA2A (p-value: 0.0003) and COL3A1 (p-value: 2.20E-05) has similar expression patterns in qRT-PCR experiments as those seen in integrated analyses of RNA-Seq data (Fig. 9A,C). GUCA2A were downregulated in both colon cancer (− 0.38-fold, *p-*value: 0.003) and rectal cancer (− 0.49-fold, *p-*value: 0.1) compared with normal tissues and COL3A1 showed upregulated expression in both colon cancer (5.58-fold, *p-*value: 0.0001) and rectal cancer (14.29-fold, *p-*value: 0.0004) compared with normal tissues (Fig. 10).

## Discussion

The most common type of gastrointestinal cancer is CRC^57^ and furthermore there are difficulties to the traditional colonoscopy diagnosis of CRC^58^. The best biomarkers are non-invasive, specific, inexpensive, sensitive, dependable and repeatable^59^. Consequently, it’s important to find a significant biomarker for CRC. Intestinal diseases such as intestinal polyps^60^ and inflammatory bowel disease^61^, which can potentially progress to cancer might display symptoms that are similar to those of CRC. Numerous research has concentrated on the pathology and mechanism of CRC although the exact mechanisms is still mostly unknown. To address the critical need for early-stage diagnostic CRC biomarkers and to investigate into the genes associated with pathogenesis we combined RNA-Seq data sets to find that 5037 genes were differently expressed between cancer tissues and normal tissues. In the second step, we investigated the upregulated gene (COL3A1) and downregulated gene (GUCA2A) in the tumor and normal samples expression profile in CRC patients using RNA-Seq data and real-time PCR validation. We performed GO and KEGG pathway enrichment analyses using enrichR package^41^. Most down-regulated genes had functions that are integral component of plasma membrane. This is consistent with the concept that pathogen avoidance and acid–base balance maintenance depend on the integral cell membrane^62,63^. The largest proportion of up-regulated genes was mainly involved in the mRNA transport, mRNA splicing, via spliceosome, intracellular non-membrane-bounded organelle and RNA binding were closely related to the development and growth of cancer^64,65^. Some KEGG pathways such as nitrogen metabolism, mineral absorption and pancreatic secretion were also linked to the pathogenesis of CRC^66^. Nitrogen is an essential biomolecule in humans and regulates cellular metabolism that related to immune functions^67^. According to the findings of the GO and KEGG enrichment studies the DEGs were closely related to the development and incidence of CRC.

Studies on Guanylate cyclase activator 2A (GUCA2A) are limited and the mechanisms are still not sufficiently understood. Guanylate cyclase activator 2A (GUCA2A) a peptide hormone secreted by gut epithelial cells, regulates guanylate cyclase 2C (GUCY2C) signaling in the autocrine and paracrine systems^68^. In more than 85% of tumors GUCA2A mRNA and protein loss is one of the most prevalent gene losses in CRC^69^. Tumor cells undergo transformation, hyper proliferation and genomic instability when the GUCY2C receptor is silenced^70,71^. Based on Samadi et al.^72^ GUCA2A is the most critical therapeutic target for all stages of colorectal cancer. Using survival analysis and ROC curve examination in CRC we identified possible prognostic and diagnostic biomarkers in this present research. According to Jalali et al.^73^ patients’ survival rate was considerably influenced by reduced levels of GUCA2A and it could potentially be utilized as a biomarker to determine a patient’s prognosis for colon cancer. Following that, ROC Curve analysis revealed that GUCA2A had the most significant AUC values and could potentially be used as a diagnostic biomarker. With the exception of a bioinformatic analysis that suggests an excellent prognosis for patients with colon cancer, there is currently insufficient research supporting its diagnostic utility for CRC patients^73^. These results are consistent with the findings of Zhang et al.^74^ which showed that COAD patients with lower GUCA2A expression levels comparison with patients with greater expression levels had a considerably shorter OS. Bashir et al. revealed that in pathophysiological circumstances a low level of GUCA2A silences the tumor inhibitory receptor GUCY2C and causes microsatellite instability in tumors^75^. Loss of GUCA2A has been seen in CRC and inflammatory bowel disease and may be related to the disturbance of intestinal homeostasis^76^. Zhang et al.^74^ showed that the expression level of GUCA2A in the colorectal cancer tissues decreased compared to healthy tissues, which is consistent with our study’s experimental results. Liu et al.^77^ used analysis of the TCGA database revealed that the expression of GUCA2A and GUCA2B was significantly downregulated in CRC tissues, which is consistent with our results. As reported by Ershov et al.^78^ the expression of GUCA2A was considerably downregulated in CRC tissues, which is consistent with our findings. According to Xu et al.^79^, GUCA2A expression level in colorectal cancer tissues were lower than in healthy tissues, which is consistent with the experimental findings from our investigations. These results suggest that GUCA2A, GUCA2B and GUCY2C may play a role in critical biological functions such as intestinal fluid management, inflammatory mediation and CRC development. However, we were unable to discover any correlation between GUCA2A expression and clinicopathological characteristics in CRC patients. Insufficient numbers of samples might account for that.

Collagen type I in connective tissue is made up of two molecules, COL1A1 and COL1A2, which are mostly produced by fibroblasts. Together with type I collagen, type III and type V collagen are present in connective tissue as alpha-1 chains known as COL3A1 and COL5A1^80,81^. The cysteine-rich acidic matrix-associated protein, encoded by SPARC, is a critical protein for ECM remodeling. It regulates how cells interact with the ECM by binding to fibronectin and collagen^82^. According to the previous studies collagen may help CRC metastasis and stemness^83^. CRC carcinogenesis is associated to abnormal COL12A1 expression^84^. According to a study COL1A1, an important collagen type I component was overexpressed in a number of tumor tissues and increased metastasis in CRC^85^. Zhao et al. revealed that COL1A1, COL1A2, COL3A1, COL5A1, and FN1 was significantly upregulated in gastric cancer patient samples^86^. Additionally, Mortezapour et al.^87^ using datasets from TCGA-COAD reported that MMP9, SERPINH1, COL1A1, COL1A2, COL5A1, COL5A2, and SPARC were significantly increased in colorectal cancer tissues compared to healthy tissues, which is in keeping with the finding of our study. Furthermore to gastric cancer, research results from several researchers have revealed that many collagen-encoding genes, including COL1A2 and COL3A1 had higher expression levels in pancreatic cancer^88^, thyroid cancer^89^ and esophageal cancer^90^. Dibdiakova et al.’s^91^ results demonstrated that COL3A1 expression was significantly higher in CRC tissues compared to normal tissues, are consistent with the results of experiments from our findings. According to Tang et al.^56^ COL3A1 has been demonstrated to be overexpressed in stage IV colorectal cancer and to be significantly downregulated in lung metastasis samples compared to liver metastasis. Li et al.^92^ indicated that COL3A1 expression was significantly higher in CRC patients in comparison to normal tissues, which agrees with our results. According to Wu et al.^84^ colon cancer tissues had significantly higher levels of COL3A1 expression than healthy tissues, which is in agreement with the experimental findings of our investigation. Wang et al.^93^ revealed that the expression level of COL3A1 was significantly increased in tumor tissues as when compared with normal tissues, which is accordance with the experimental results of our study. Additionally, these researchers demonstrated that COL3A1 expression was substantially correlated with age, sex, stage, T stage, Dukes stage, tobacco use, recurrence, and survival status in various cohorts of patients with CRC. While it was discovered that the grade, stage, and T stage of CRC patients were related to the overexpression of the COL3A1 protein. These findings indicated that COL3A1 could be useful as a molecular signature for CRC. Additionally, in our study, there was no correlation between the level of COL3A1 expression in colon and rectum cancers and the clinical and pathological characteristics of the CRC patients. However, the limited sample size may be responsible for this. COL3A1 demonstrated an excellent diagnostic potential for differentiating between malignant and normal tissues, according to the ROC Curve. However, experimental confirmation of this gene showed a considerable increase in CRC tissues as compared with normal tissues, suggesting its potential as a prognostic biomarker. In our PPI analysis of the top 80 significantly DEGs, two of the significant hub proteins GUCA2A and COL3A1 were also shown to have a significant role in CRC.

CLCA4 has the ability to inhibit the growth and invasion of CRCs^94,95^. Zhao et al. found that CLCA4 expression was low in CRC patients^96^, which is consistent with our bioinformatics results. Additionally, based on Li et al.^97^ CLCA4 expression was significantly decreased in CRC patients’ tissues when compared to normal tissues, which is consistent with our findings. For both colon and rectal cancer, CLCA1 has been approved as a diagnostic and prognostic biomarker^98^. Li et al.^99^ identified that CLCA1 inhibits the Wnt/beta-catenin signaling pathway and the epithelial-mesenchymal transition (EMT) to play a significant function as an inhibitor of tumor growth in CRC. Yang et al.^100^ found that the expression of CLCA1 and CLCA4 was considerably down-regulated in CRC patients in comparison with healthy tissues, which is in keeping with our research. The proliferation and invasion of colon cancer cells can be inhibited by the overexpression of AQP8 a member of the aquaporin family^101^. Consequently, Zhang et al.^102^ reported that the expression level of AQP8 was substantially reduced in CRC tissues comparable to normal tissues, which is consistent with our results and having high levels of AQP8 was related to increased survivability in patients suffering from CRC. One of the key transporters that excretes oxalate is SLC26A6, which is mostly expressed in the small intestine comparison SLC26A3 can regulate oxalate absorption in ileum, cecum and colon^103^. The SLC26A3 mutation was associated to inflammatory bowel diseases^104^, thus mutation of intestinal SLC26A3 may be a risk factor for CRC. Lin et al.^105^ showed that up-regulation of SLC26A3 prevented CRC growth and metastasis whereas down-regulation of SLC26A3 accelerated CRC progression by modifying the level of IκB expression, in addition, these researchers discovered that SLC26A3 expression was significantly decreased in tumor tissues as compared with normal tissue, which is consistent with our study. Samadi et al.^72^ reported that the most significant therapeutic targets for all stages of CRC are CLCA1, AQP8, CLCA4 and SLC26A3. A number of previous research have demonstrated that the secreted protein CXCL8 functions with its receptors, CXCR1 and CXCR2 to promote the development of several cancers including breast cancer^106^, prostate cancer^107^ and CRC^108^. The CXCL8 gene is upregulated in CRC tissue and correlated with the development of CRC^109^, which is consistent with our bioinformatics results. According to research by Xia et al., high levels of CXCL8 expression are substantially related to poor overall survival, tumor stage, lymphatic and liver metastasis^110^. Fisher et al. show that inhibiting the CXCL8-CXCR1 pathway can reduce the tumorigenicity that develops in CRC stem cells^111^ therefore, more research is necessary to identify the accurate association between CXCL8 expression and the CRC. TGFBI promotes tumor development in CRC and its silencing prevents both in vivo tumor growth and in vitro angiogenesis^112^. Its expression is increased in esophageal squamous cell carcinoma^113^, gastric cancer^114^ and bladder cancer^115^. In contrast to normal tissues, less TGFBI expression can be seen in some cancers, such as lung cancer^116^ and breast cancer^117^. Gao et al. used analysis of the TCGA data indicated that the expression of TGFBI was dramatically overexpressed in colon cancer tissues^118^, which is consistent with our bioinformatics results. According to researchers, ACTG1 which is upregulated in cancer, increases the progression of hepatocellular carcinoma^119,120^. Ming et al. found that the expression of ACTG1 was considerably upregulated in colon adenocarcinoma based on genome-scale CRISPR-Cas9 knockout (GeCKO) screening and TCGA-COAD data^121^, which is consistent with the results of our bioinformatics research. In patients with CRC abnormal APOA4 expression was related to 8q24 oncogenic SNPs and revealing that this protein could contribute to CRC proliferate^122^. In accordance with the results we obtained, Ahn et al.^123^ determined that APOA4 levels across all CRC stages significantly decreased in compared to healthy samples, and Voronova et al.^124^ identified that APOA4 expression levels were considerably lower in tumor tissues than in normal tissue. The present research is used as an initial test for future studies with the goal to validate these particular genes as diagnostic biomarkers. More analysis and research on these specific genes might lead to novel therapeutic targets for CRC.

In this study, suggestions for future studies are presented. First: Examining the expression of GUCA2A and COL3A1 in blood samples, serum, colorectal cancer cell lines, their role with using overexpression and knock-down methods of genes. Second: Long-term study of changes in the expression of GUCA2A and COL3A1 genes in a larger number of patients with colorectal cancer. Third: Examining related bioinformatics studies on a larger scale and finding related genes and clinical examination on them. Fourth: Research on the mRNAs, miRNAs and proteins related to these genes in order to produce liquid biopsy tests that can replace surgical tests for diagnosis.

## Conclusion

An opportunity to create an innovative therapeutic approach and have an essential effect with respect to enhancing the final outcome of CRC patients might derive from the identification of the GUCA2A and COL3A1 accountable for CRC. To improve our knowledge and enhance caring for patients in colorectal cancer, additional research into of these genes and their functions in CRC is crucial.

### Supplementary Information

Supplementary Figures.

Supplementary Tables.

## Data Availability

Publicly available datasets were analyzed in this study. These data can be found at PRJNA562898, PRJNA691157, PRJNA778353 and PRJNA603221 from Sequence Read Archive (SRA, https://www.ncbi.nlm.nih.gov/sra). All other data supporting the findings of this study are available within the article and the Supporting Information or from the corresponding author upon reasonable request.
