# Supplementary material for: Identification of GUCA2A and COL3A1 as prognostic biomarkers in colorectal cancer by integrating analysis of RNA-Seq data and qRT-PCR validation
Source: Sci Rep. 2023 Oct 10;13:17086. doi: 10.1038/s41598-023-44459-y (PMC10564945; doi:10.1038/s41598-023-44459-y)
Supplement: Supplementary file 1 — Supplementary Figures. [file 41598_2023_44459_MOESM1_ESM.docx]

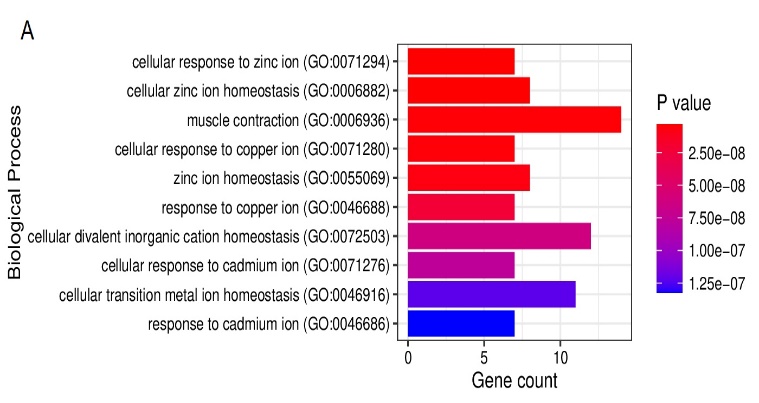

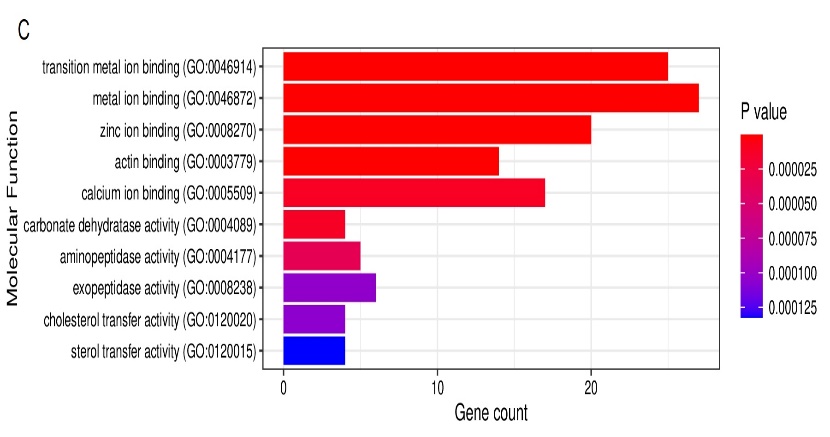

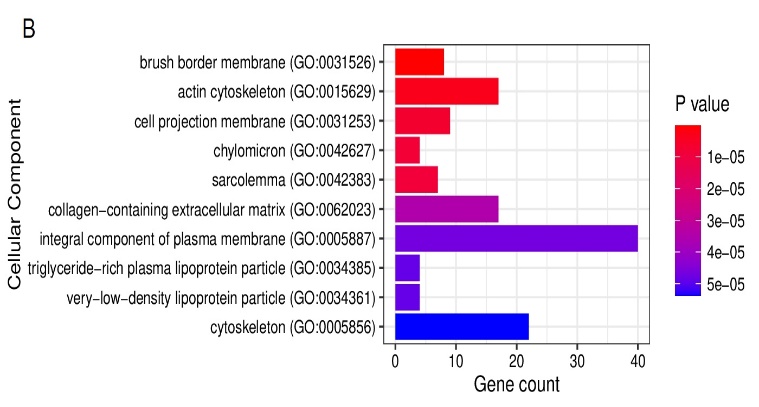
**Supplementary Information (Figures)**

**Figure S1. GO enrichment of downregulated genes.** (**A**) GO enrichment of biological process. (**B**) GO enrichment of cellular components. (**C**) GO enrichment of molecular function. The significant enriched GO terms were based on *p* value < 0.05.


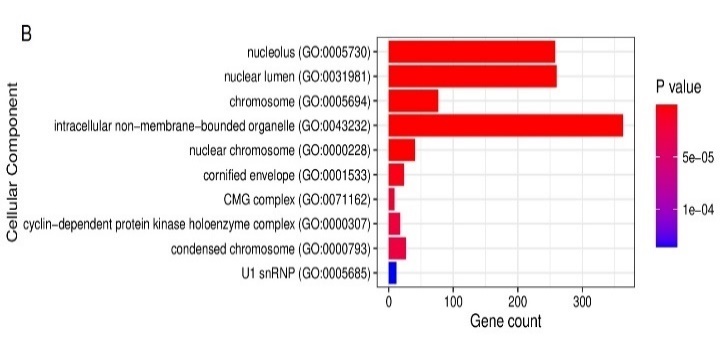

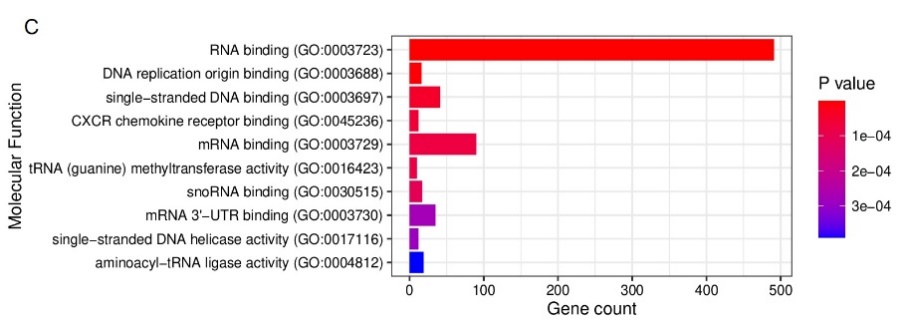

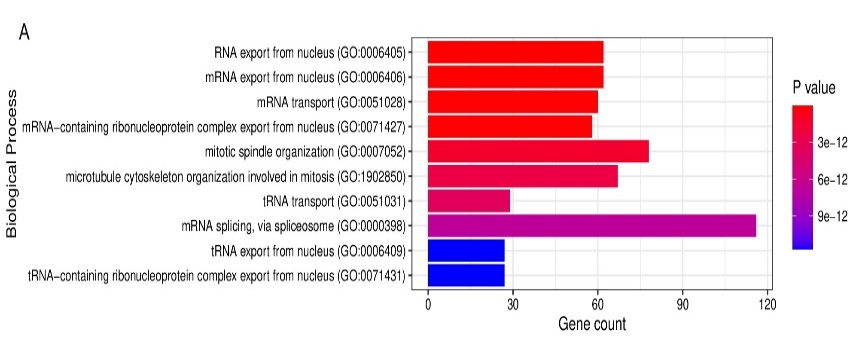


**Figure S2. GO enrichment of upregulated genes.** (**A**) GO enrichment of biological process. (**B**) GO enrichment of cellular components. (**C**) GO enrichment of molecular function. The significant enriched GO terms were based on *p* value < 0.05.


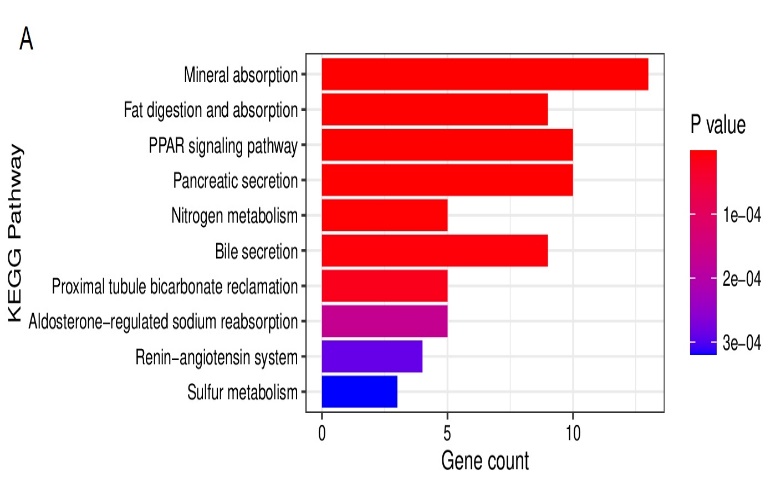

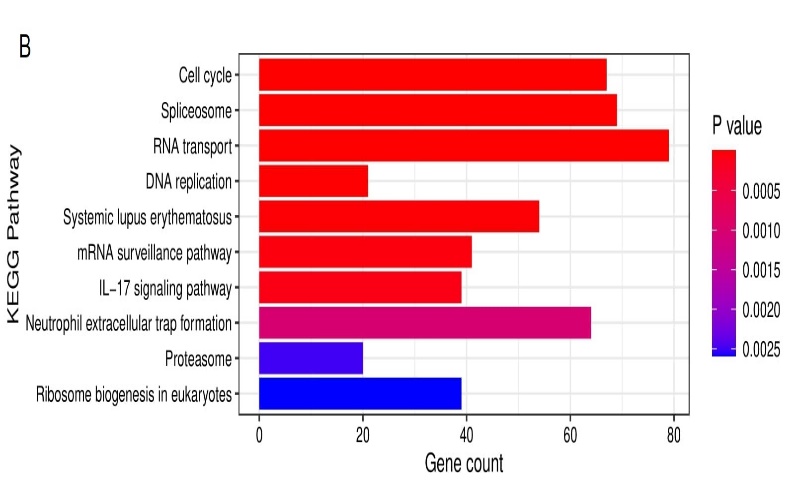


**Figure S3. KEGG pathway enrichment analysis.** (**A**) KEGG Pathway enrichment of downregulated genes. (**B**) KEGG Pathway enrichment of upregulated genes. The significant enriched KEGG pathway were based on *p* value < 0.05.
